# Patient–physician communication about cancer-related fatigue: a survey of patient-perceived barriers

**DOI:** 10.1007/s00432-023-05555-8

**Published:** 2024-01-25

**Authors:** Marlena Milzer, Anna S. Wagner, Martina E. Schmidt, Imad Maatouk, Silke Hermann, Cancer Registry of Baden-Württemberg, Senta Kiermeier, Karen Steindorf

**Affiliations:** 1 Division of Physical Activity, Prevention and Cancer, German Cancer Research Center (DKFZ), Heidelberg, Germany; 2 Medical Faculty, University of Heidelberg, Heidelberg, Germany; 3 Section of Psychosomatic Medicine, Psychotherapy and Psychooncology, Department of Internal Medicine II, Julius-Maximilian-University, Würzburg, Germany; 4 Epidemiological Cancer Registry of Baden-Württemberg, German Cancer Research Center (DKFZ), Heidelberg, Germany; 5 National Center for Tumor Diseases (NCT), NCT Heidelberg, a partnership between DKFZ and University Medical Center Heidelberg, Heidelberg, Germany

**Keywords:** Cancer-related fatigue, Patient participation, Patient–physician communication, Structural barriers, Supportive care

## Abstract

**Purpose:**

Cancer-related fatigue is a subjective, distressing, and common sequela of cancer which is often disregarded and underdiagnosed. Fatigue is assessed by self-report requiring communication between patient and physician. In this study, we investigated the patients’ perspective on the patient–physician communication about fatigue.

**Methods:**

On average five months after diagnosis 1179 cancer patients, recruited in Germany, completed a survey as part of the LIFT project. The survey included questions on sociodemographic data, fatigue, depression, fatigue management, patient–physician communication, and communication barriers. Data were analyzed descriptively and using logistic regression analyses.

**Results:**

Half of the participants reported that their physician had never asked them whether they felt exhausted. Patients undergoing chemo-, radio-, or immunotherapy were more likely to be asked about fatigue, while older age and major depression decreased the likelihood. Sixty-four percent of the patients felt impeded by communication barriers. Common barriers were not knowing who to turn to for fatigue (39%), time constraints (31%), and the fear of being perceived as weak (22%). Almost half of the participants indicated that their physicians were not appreciative and did not deal adequately with fatigue-related questions.

**Conclusion:**

This study revealed gaps in the patient–physician communication regarding cancer-related fatigue. Contrary to guideline recommendations a minority of physicians addressed fatigue. On the other hand, cancer patients felt reluctant to bring up this topic due to structural barriers and fears. Physicians should routinely address fatigue and adopt a communication style which encourages patients to likewise state their symptoms and raise their questions.

**Trial registration:**

Clinicaltrials.gov, identifier: NCT04921644. Registered in June 2021.

## Introduction

Cancer-related fatigue (CRF) refers to a ‘distressing, subjective sense of physical, emotional and/or cognitive tiredness or exhaustion related to cancer or cancer treatment that is not proportional to recent activity and interferes with usual functioning’ (National Comprehensive Cancer Network 2023). As shown by a large-scale multicenter study, almost half of the cancer patients undergoing treatment show moderate to severe CRF levels (Wang et al. 2014). CRF may persist years after diagnosis, with prevalences differing between cancer types (Schmidt et al. 2020). Sociodemographic characteristics as well as disease-related factors (e.g., cancer type, cancer treatment, stage, performance status) seem to affect CRF (Andrykowski et al. 2005; Bower 2019; Wang et al. 2014). Furthermore, CRF is more likely to occur in individuals with a history of depression or anxiety and in individuals who are physically inactive (Kuhnt et al. 2019; Roila et al. 2019; Wang et al. 2014). However, the underlying biological mechanisms have not yet been elucidated in detail (Bower 2019).

CRF has a negative impact on quality of life and hampers the ability to carry out daily activities and return to work (Arndt et al. 2006; Behringer et al. 2016; Jung et al. 2018; Schmidt et al. 2019). Generally, non-pharmacological interventions are superior to pharmacological interventions in the treatment of CRF (Mustian et al. 2017). High-level scientific evidence, both for patients under treatment and post-treatment, is available for physical activity including yoga and for psychosocial interventions (psychoeducation, cognitive behavioral therapy) (National Comprehensive Cancer Network 2023).

The above-introduced definition of CRF as a *subjective* experience implies that CRF is most accurately captured by self-reports (National Comprehensive Cancer Network 2023). Indeed, in the absence of specific biological markers, communication between patients and healthcare professionals (HCPs) about CRF is necessary. Clinical practice guidelines for CRF recommend HCPs to initiate communication about CRF by conducting a systematic screening and offering education about CRF (Fabi et al. 2020; National Comprehensive Cancer Network 2023). More precisely, cancer patients should be routinely and regularly screened for CRF using valid and reliable instruments with established cut-offs, such as a numeric rating scale ranging from 0 (‘*no fatigue’*) to 10 (‘*worst fatigue imaginable’*) (Fabi et al. 2020; National Comprehensive Cancer Network 2023). Furthermore, it is suggested to inform all cancer patients and their caregivers about characteristics of CRF and about general strategies for coping with CRF (e.g., physical activity and energy conservation) (National Comprehensive Cancer Network 2023).

However, previous studies indicated that the recommendations are not yet implemented in clinical practice, presumably due to HCP-related, patient-related, and structural barriers (Berger et al. 2015; Berger and Mooney 2016; Schmidt et al. 2021; Smith et al. 2019). These shortcomings in CRF management lead to knowledge gaps and helplessness in patients, and may prevent efficient self-management of CRF (Schmidt et al. 2022).

If HCPs do not follow guideline recommendations by addressing CRF, do patients themselves initiate communication about CRF in medical consultations or what might impede them from doing so? Previous research has shown that patients with chronic conditions commonly report barriers to participating in medical consultations (Henselmans et al. 2015). These include the fear of being bothersome, a lack of time and remembering one’s own agenda and questions only after the medical appointment (Henselmans et al. 2015). Studies explicitly targeting communication between patient and HCPs in CRF management revealed that a lack of awareness of available treatment options and the belief that CRF is an inevitable consequence of cancer (treatment) hinder patients from addressing CRF with HCPs (Passik et al. 2002; Shun et al. 2009; Stone et al. 2000). Since the just mentioned studies were conducted before CRF-specific guidelines of major cancer organizations had been published and were mainly based on relatively small sample sizes, our study aimed to assess the patients’ perspective on current patient-physician communication about CRF. More specifically, we wanted to (1) clarify to what extent physicians initiate communication about CRF by providing information and screening, (2) investigate how patients perceive the quality of CRF-related communication with physicians, (3) identify communication barriers, and (4) explore factors associated with the perception of barriers. From this, we will derive suggestions for reducing communication barriers with the aim of enhancing participatory communication of patients as a core skill of self-management as well as HCP-driven CRF management (Howell et al. 2022).

## Methods

### Design and participants

This observational study among cancer patients was conducted as part of the LIFT project (Clinicaltrials.gov, identifier: NCT04921644). The LIFT project pursues the goal of assessing current CRF management in Germany from the patients’, HCPs’, and institutional perspectives to identify targets for improvements. Recruitment for the patient survey was carried out between August 2021 and September 2022. Inclusion criteria were as follows: (1) ≥ 18 years of age, (2) (newly) diagnosed primary tumor, (3) recent or current chemotherapy, radiotherapy, hormone therapy, targeted or immune therapy, (4) ability to understand and follow the study protocol. Patients were randomly sampled from the Epidemiological Cancer Registry of Baden-Wuerttemberg, Germany, stratified by cancer entity. The Trust Center of the Cancer Registry invited patients via postal mail to participate in the study. Patients who decided to participate had two options: they could either directly access the online survey with the individual access key contained in the letter or provide consent to the Trust Center to transfer their contact data to the LIFT study team in order to receive the paper–pencil version of the questionnaire.

### Procedures and measures

After having provided informed consent, patients filled out either the online or the paper–pencil survey. Completing the questionnaire took patients approximately 45 min.

Severity of CRF, including interference with daily life, was assessed with the Brief Fatigue Inventory (BFI) (Mendoza et al. 1999). By calculating the arithmetic mean over the 9 items, the global BFI score was obtained, with values < 1 being classified as no fatigue, values ranging from 1 to 3 as mild, from 4 to 6 as moderate, and from 7 to 10 as severe fatigue. Furthermore, to multidimensionally measure CRF, the European Organisation for Research and Treatment of Cancer (EORTC) fatigue module (EORTC QLQ-FA12) was used (Weis et al. 2017). The EORTC-QLQ-FA12 consists of 12 items that are allocated to the physical, emotional and cognitive domains of fatigue (Weis et al. 2017, 2019). The questionnaire reveals excellent psychometric properties (Campbell et al. 2022). Depression symptoms were measured with the Patient Health Questionnaire (depression module, PHQ-9) showing good reliability and validity (Arroll et al. 2010; Kroenke et al. 2001; Löwe et al. 2004). Sum scores below 5 indicate the absence of a depressive disorder (PHQ score = 0), while sum scores between 5 and 10 correspond to a minor depression (PHQ score = 1) and scores above 10 to a major depression (PHQ score = 2). The German version of the short form of the Illness-specific Social Support Scale (‘Skalen zur sozialen Unterstützung bei Krankheit’—SSUK-8) was used to assess social support (Ullrich and Mehnert 2010). The questionnaire is composed of eight items which have to be rated on a 5-point Likert scale ranging from 0 (‘*never’*) to 4 (‘*always’*). Four items refer to the subscale ‘*positive support*’, the other four items make up the subscale ‘*detrimental interaction*’ (Revenson et al. 1991; Ullrich and Mehnert 2010).

Screening for CRF was assessed with two questions. First, participants had to indicate if and when they were asked by their treating physicians whether they felt exhausted. In a next step, they should state if and when they were asked to rate CRF by means of a scale or a questionnaire. Further, patients should rate on a 4-point Likert scale ranging from 0 (‘*not informed at all’*) to 3 (‘*well informed’*) how well they were informed about characteristics, causes and treatment options of CRF. Moreover, agreement to the statement ‘*My caregivers were well informed about fatigue.*’ should be rated on a 4-point Likert scale with the scale endpoints 0 (‘*strongly disagree)* to 3 (‘*strongly agree’*) to investigate if, following guideline recommendations, caregivers were involved in education about CRF. Likewise, patients’ perceived quality of communication with physicians about CRF was assessed with five statements (e.g., ‘*My physicians are appreciative and understanding of my questions about CRF*’, ‘*My physicians take time to address my questions about CRF*’). To investigate barriers hindering patients from addressing CRF in medical consultations, agreement to ten statements (e.g., ‘*I am afraid of being perceived as weak and sniveling*’, *‘I do not know who to turn to for CRF’*) should be judged on a 4-point Likert scale. Items for assessing communication barriers were derived from the Fatigue Management Barriers Questionnaire (Passik et al. 2002) and adjusted to more recent findings in literature and guidelines. Clinical data of the study population (including cancer type, stage, cancer treatment, etc.) were provided by the Epidemiological Cancer Registry and transmitted in encrypted form to the LIFT study team.

### Statistical methods

Patient characteristics and data concerning CRF management, patient–physician communication, and perceived communication barriers are presented descriptively. To determine the factors associated with physicians addressing CRF (yes vs. no) or informing about CRF (yes vs. no), respectively, separate multiple logistic regression analyses were calculated. Based on univariate analyses and theoretical considerations, the categorical factors sex, educational level, German native language, metastasis, chemotherapy, radiotherapy, hormone therapy, immunotherapy, fatigue severity and depression, and the continuous variable age were included. The number of perceived barriers was determined as follows: First, each of the eight items assessing communication barriers were dichotomized. The value of 0 was assigned if participants had selected the answer options ‘*strongly disagree*’ or ‘*disagree*’, while the value of 1 was given if patients had (*strongly*) *agreed* to the statement. A sum score of the dichotomized variables was then calculated, resulting in a minimum of 0 and a maximum of 8. To explore which patient-related factors are associated with perceiving communication barriers, further multiple logistic regression analyses were performed separately for each dichotomized barrier. The following factors were included simultaneously: sex, age, educational level, chemotherapy, fatigue severity, depression, and social support (subscale ‘*positive support*’ of the SSUK-8). Likewise, a logistic regression analysis was performed for the outcome variable ‘number of communication barriers’ (no communication barrier vs. ≥ 1 communication barrier).

## Results

### Study population

**Fig. 1:**
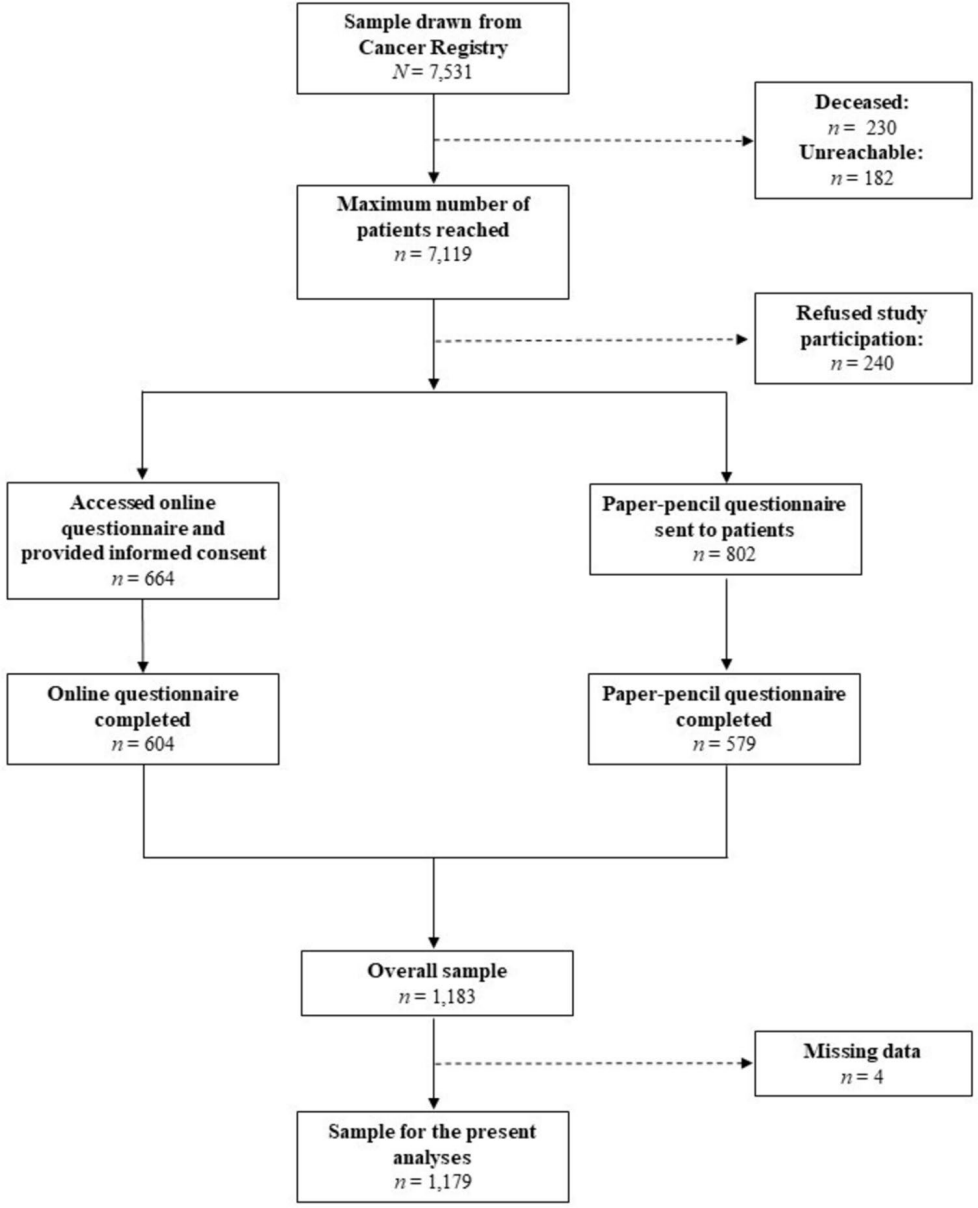
Recruitment flow

The recruitment flow is presented in Fig. 1. Between August 2021 and September 2022, a total of 7,531 cancer patients were contacted by the Cancer Registry of Baden-Württemberg, Germany, of which 230 were deceased and 182 were unreachable due to incorrect address data. Of the remaining 7,119 patients, 240 actively refused transfer of data to the study center. Of the 802 patients who consented to be sent a paper–pencil questionnaire, a total of 579 patients completed the questionnaire, leading to a return rate of 72.2%. Additionally, 664 patients accessed the online survey and provided informed consent, 604 of these (91.0%) completed the online questionnaire. Thus, the overall sample consisted of 1,183 cancer patients. Due to missing data, four patients had to be excluded for the present analyses, resulting in the final sample of 1,179 participants. Descriptive statistics of the study population are displayed in Table 1. The mean age of the study population was 63 years (SD = 12.5) and 56% were female. Mean time since diagnosis was 5.2 months (SD = 1.5). The most common cancer types in this sample were breast cancer (26%), colorectal cancer (16%) and prostate cancer (14%). Metastases were reported by 19% of the study population. Seventy-four percent had received tumor surgery, 43% had either completed or were currently receiving chemotherapy. The majority of the participants reported CRF symptoms with 42% being classified as mild, 30% as moderate and 7% as severe. According to the PHQ-9, the diagnostic criteria for a major depression were met in approximately 10% of the participants.

**Table 1:** Sociodemographic and medical characteristics of the study population (*N* = 1179)

Characteristic	Mean	Standard deviation
Age [years]	63.3	12.5
Time since diagnosis [months]	5.2	1.5

Characteristic	Number of cases^*a*^	%
Sex		
Female	659	55.9
Male	520	44.1
Educational level^b^		
Lower	734	62.8
Higher	434	37.2
German native language		
Yes	1062	90.9
No^c^	106	9.1
Current work status^d^		
Currently not working	915	79.2
Currently working	240	20.8
Cancer type		
Breast	308	26.1
Colorectal	193	16.4
Prostate	166	14.1
Lung	92	7.8
Malignant melanoma	81	6.9
Lymphoma	54	4.6
Pancreas	43	3.6
Stomach	40	3.4
Leukemia	38	3.2
Thyroid gland	36	3.1
Bladder or kidney	34	2.9
Gynecological tumors except breast	32	2.7
Liver	26	2.2
Multiple^e^	36	3.1
Metastasis		
No	897	76.1
Yes	226	19.2
Unknown	56	4.7
UICC stage		
0	2	0.2
I	352	29.9
II	223	18.9
III	145	12.3
IV	156	13.2
Unknown	301	25.5
Comorbidities		
None	257	22.0
≥ 1	909	78.0
Surgery		
No	304	25.8
Yes	875	74.2
Chemotherapy^f^		
No	672	57.0
Yes	507	43.0
Radiotherapy^f^		
No	801	67.9
Yes	378	32.1
Hormone therapy^f^		
No	918	77.9
Yes	261	22.1
Immunotherapy^f^		
No	951	80.7
Yes	228	19.3
Targeted therapy^f^		
No	1,128	95.7
Yes	51	4.3
Fatigue severity^g^		
No fatigue	233	21.0
Mild fatigue	463	41.6
Moderate fatigue	334	30.0
Severe fatigue	82	7.4
Depression^h^		
No depression	946	84.5
Minor depression	67	6.0
Major Depression	106	9.5

Characteristic	Median	First quartile, third quartile
EORTC QLQ-FA12		
Physical fatigue	53	27, 73
Emotional fatigue	22	0, 56
Cognitive fatigue	0	0, 33

^a^Numbers in cells may not add up to total *N* = 1,179 due to missing data

^b^Lower: no degree or (lower-) secondary education degree; *Higher:* diploma qualifying for university or university degree

^c^No*:* ‘No, but I speak German fluently’ (*n* = 70), ‘No, but I understand almost everything when German is spoken’ (*n* = 18), ‘No, I have difficulty understanding and speaking German’ (*n* = 18).

^d^Currently not working: on sick leave (*n* = 297), retired (*n* = 545), unemployed (*n* = 13), homemaker (*n* = 60); Currently working: employed (*n* = 235), student/in training (*n* = 5)

^e^Multiple primary tumors at different locations

^f^No*:* Not (yet) having received or receiving the treatment; *Yes:* Treatment completed or currently ongoing treatment

^g^Referring to the Brief Fatigue Inventory (BFI) score

^h^Referring to the Patient Health Questionnaire, depression module (PHQ-9) score

### Physician-initiated communication about CRF

**Fig. 2:**
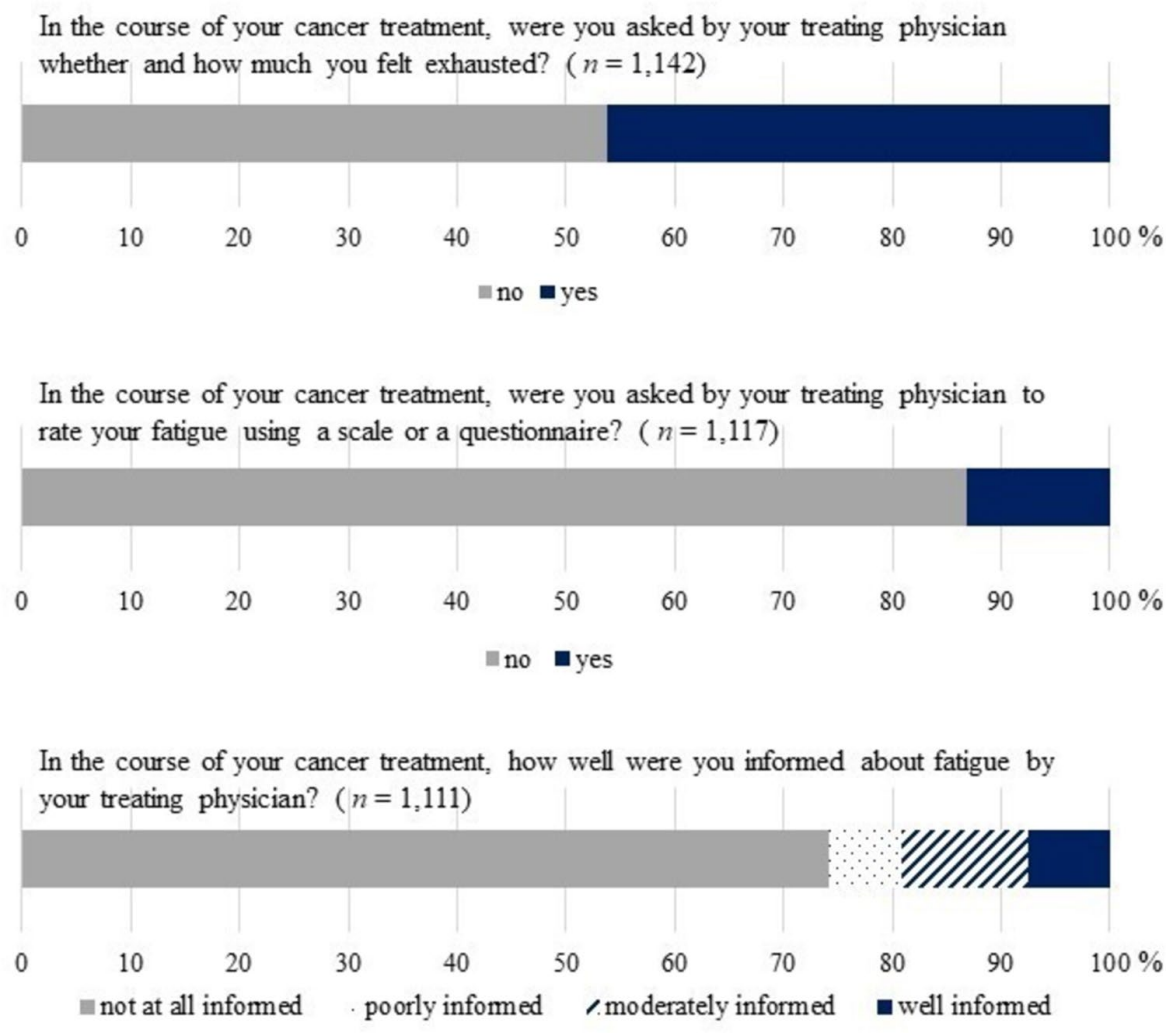
The patients’ perspective on physician-provided screening and education about cancer-related fatigue

As shown in Fig. 2, more than half of our participants (54%) reported that they have never been asked by their treating physician whether and how much they felt exhausted. Only 36 participants (3%) indicated that their physicians brought up the topic of CRF either before and repeatedly during cancer therapy. Results of the logistic regression analysis are displayed in Table 2. The logistic regression revealed chemotherapy, radiotherapy and immunotherapy as significant predictors, meaning that patients who had received or who were currently receiving the respective treatment were more likely to be asked by their treating physician about CRF (chemotherapy: OR = 2.56, 95% CI [1.90; 3.45], *p* < 0.001; radiotherapy: OR = 1.67, 95% CI [1.22; 2.31], *p* = 0.002; immunotherapy: OR = 1.54, 95% CI [1.07; 2.21], *p* = 0.020). Furthermore, higher age decreased the likelihood of being asked by physicians about CRF (*OR* = 0.98, 95% CI [0.97; 0.99], *p* < 0.001). Patients without depression were two times more likely to be asked about CRF by their treating physician than patients with major depression (OR = 0.50, 95% CI [0.29; 0.85], *p* = 0.011).

**Table 2:** Logistic regression results on factors associated with patients reporting that their physicians addressed fatigue and informed about fatigue

	Physicians addressed fatigue^a^			Physicians informed about fatigue^b^		
	(*N* = 1005)			(*N* = 984)		
	OR	CI	*p*	OR	CI	*p*
Sex						
Male	Reference			Reference		
Female	0.96	(0.72; 1.28)	0.760	0.88	(0.63;1.22)	0.439
Age						
Per one year	0.98	(0.97;0.99)	** < 0.001**	0.98	(0.97;0.99)	**0.001**
Educational level^c^						
Lower	Reference			Reference		
Higher	1.04	(0.78; 1.37)	0.802	1.03	(0.75;1.41)	0.875
German native language						
No	Reference			Reference		
Yes	0.69	(0.43;1.11)	0.125	0.45	(0.28;0.73)	**0.001**
Metastasis						
No	Reference			Reference		
Yes	1.30	(0.92;1.85)	0.142	1.13	(0.76;1.67)	0.551
Unknown	1.60	(0.77;3.34)	0.207	1.38	(0.65;2.95)	0.402
Chemotherapy^d^						
No	Reference			Reference		
Yes	2.56	(1.90;3.45)	** < 0.001**	2.03	(1.45;2.85)	** < 0.001**
Radiotherapy^d^						
No	Reference			Reference		
Yes	1.67	(1.22;2.31)	**0.002**	1.48	(1.03;2.12)	**0.033**
Hormone therapy^d^						
No	Reference			Reference		
Yes	1.41	(0.98;2.03)	0.064	1.29	(0.86;1.94)	0.223
Immunotherapy^d^						
No	Reference			Reference		
Yes	1.54	(1.07;2.21)	**0.020**	1.40	(0.95;2.06)	0.088
Fatigue severity^e^						
No fatigue	Reference			Reference		
Mild fatigue	0.93	(0.65;1.32)	0.671	0.71	(0.47;1.06)	0.092
Moderate fatigue	1.12	(0.75;1.66)	0.592	0.92	(0.59;1.43)	0.701
Severe fatigue	1.36	(0.70;2.65)	0.371	0.82	(0.38;1.74)	0.597
Depression^f^						
No depression	Reference			Reference		
Minor depression	1.28	(0.72;2.27)	0.400	1.38	(0.75;2.55)	0.299
Major depression	0.50	(0.29;0.85)	**0.011**	0.54	(0.28;1.02)	0.058

Bold values indicate statistical significance at the *p* < 0.05 level

^a^Patient reported to be asked by a physician whether and how much he/she felt exhausted

^b^Patient reported to be informed by a physician about fatigue

^c^Lower: no degree or (lower-) secondary education degree; Higher*:* diploma qualifying for university or university degree

^d^No*:* Not (yet) having received or receiving the treatment*;* Yes*:* Treatment completed or currently ongoing treatment

^e^Referring to the Brief Fatigue Inventory (BFI) score

^f^Referring to the Patient Health Questionnaire, depression module (PHQ-9) score

Contrary to the guideline recommendation, a minority of 13% of the sample was asked by their treating physicians to rate fatigue using a scale or a questionnaire (see Fig. 2). Additionally, 74% of the participants were not at all informed about CRF by their treating physician in the course of cancer treatment. About 8% stated to be well informed about the topic. A small percentage of participants (6%) rather or fully agreed that their caregivers were well informed about CRF. The logistic regression analysis showed that recent or current chemotherapy and radiotherapy were associated with an increased likelihood of being informed by physicians about CRF (chemotherapy: OR = 2.03, 95% CI [1.45; 2.85], *p* < 0.001; radiotherapy: OR = 1.48, 95% CI [1.03; 2.12], *p* = 0.033). Higher age and German native language, on the other hand, decreased the likelihood of being informed about CRF (age: OR = 0.98, 95% CI [0.97; 0.99], *p* = 0.001; German native language: OR = 0.45, 95% CI [0.28;0.73], *p* = 0.001). The corresponding results are presented in Table 2.

### Patient-perceived communication barriers

The majority of the patients (64%) reported ≥ 1 barrier impeding them from addressing exhaustion in medical consultations. While 18% perceived just one communication barrier, 17% felt impeded by ≥ 5 barriers. Of those perceiving exactly one communication barrier, the most frequently affirmed barriers were ‘*There is/was no opportunity to address fatigue*’, ‘*I do not know who to turn to for fatigue*’ and ‘*I am afraid of being perceived as weak and sniveling*’. Overall, 39% of the participants agreed to the statement ‘*I do not know who to turn to for fatigue*’ and 27% to ‘*I think that my physicians consider fatigue not to be important*’. Moreover, 26% of the study population agreed that their physicians did not have enough knowledge about fatigue. The least frequently named barrier was ‘*I am afraid that this [addressing my exhaustion] could lead to a dose reduction or to a discontinuation of cancer therapy*’ (see Table 3).

**Table 3:** Perceived barriers impeding patients to address their exhaustion with their physicians

Communication barrier	Strongly disagree		Disagree		Agree		Strongly agree	
	*n*	%	*n*	%	*n*	%	*n*	%
I am afraid of being perceived as weak and sniveling. (*N* = 1122)	657	58.6	215	19.2	179	16.0	71	6.3
I am afraid that this could lead to a dose reduction or to a discontinuation of cancer therapy. (*N* = 1107)	809	73.1	207	18.7	71	6.4	20	1.8
There is/was no opportunity. (*N* = 1085)	510	47.0	187	17.2	246	22.7	142	13.1
I think that my physicians do not have time to talk about this issue. (*N* = 1093)	483	44.2	272	24.9	250	22.9	88	8.1
I think that my physicians consider fatigue not to be important. (*N* = 1061)	457	43.1	320	30.2	224	21.1	60	5.7
I think that there are no treatments for fatigue. (*N* = 1044)	460	44.1	347	33.2	182	17.4	55	5.3
I do not know who to turn to for fatigue. (*N* = 1064)	435	40.9	216	20.3	263	24.7	150	14.1
My physicians do not have enough knowledge about fatigue. (*N* = 1001)	401	40.1	344	34.4	204	20.4	52	5.2
Number of communication barriers^a^ (*N* = 1096)	*n*	%						
0	394	35.9						
1^b^	192	17.5						
2	122	11.1						
3	103	9.4						
4	100	9.1						
≥ 5	185	16.9						

^a^Based on dichotomized variable with 0: fully disagree, rather disagree; 1: rather agree, fully agree

^b^Of those, the most frequently perceived barriers were: ‘There is/was no opportunity’ (31.3%); ‘I do not know who to turn to for fatigue (20.8%); ‘I am afraid of being perceived as weak and sniveling’ (14.6%)

The results of the logistic regression analyses are depicted in Table 4. Significant associations between educational level, fatigue severity, depression and perceiving ≥ 1 communication barrier were revealed, with higher education (*OR* = 1.34, 95% CI [1.00; 1.80], *p* = 0.049), a more pronounced CRF severity (mild fatigue—BFI score 1 vs. no fatigue: *OR* = 2.21, 95% CI [1.57; 3.11], *p* < 0.001) and major depression (*OR* = 3.54, 95% CI [1.50; 8.37], *p* = 0.004) increasing the likelihood of perceiving ≥ 1 communication barrier. When separately considering the communication barriers, CRF severity was significantly associated with each individual communication barrier (e.g., ‘*fear of being perceived as weak*’, mild fatigue: OR = 2.05, 95% CI [1.17; 3.58], *p* = 0.012). Furthermore, female sex (OR = 1.94, 95% CI [1.36; 2.75], *p* < 0.001) and depression (major depression: *OR* = 2.50, 95% CI [1.46; 4.29], *p* < 0.001) increased the likelihood of perceiving the barrier ‘*fear of being perceived as weak*’ whereas chemotherapy (OR = 0.48, 95% CI [0.34; 0.68], *p* < 0.001) and higher age decreased the likelihood (OR = 0.97, 95% CI [0.95; 0.98], *p* < 0.001). The likelihood of feeling impeded by the communication barrier ‘*physicians consider fatigue not important*’ was increased by higher CRF severity (mild CRF: OR = 1.90, 95% CI [1.16; 3.11], *p* = 0.010) and depression (major depression: OR = 1.90, 95% CI [1.11; 3.25], *p* = 0.019) and decreased by chemotherapy (OR = 0.57, 95% CI [0.41; 0.80], *p* < 0.001) as well as positive social support (OR = 0.93, 95% CI [0.89; 0.98], *p* = 0.003). Individuals with more severe CRF (mild CRF: OR = 2.06, 95% CI [1.36; 3.14], *p* < 0.001) or higher depression scores (major depression: OR = 4.87, 95% CI [2.65; 8.98], *p* < 0.001) were more likely to perceive the barrier ‘*not knowing who to turn to for fatigue*’. Moreover, higher education (OR = 0.73, 95% CI [0.54; 0.99], *p* = 0.040) as well as more positive social support (OR = 0.95, 95% CI [0.91; 0.99], *p* = 0.022) decreased the likelihood.

**Table 4:** Results of the separate multiple logistic regression analyses on factors associated with the perception of communication barriers

	Fear of being perceived as weak			Fear of dose reduction or discontinuation of therapy			No opportunity to discuss fatigue		
	(*N* = 1008)			(*N* = 997)			(*N* = 978)		
	OR	CI	*p*	OR	CI	*p*	OR	CI	*p*
Sex									
Male	Reference			Reference			Reference		
Female	1.94	(1.36;2.75)	** < 0.001**	1.05	(0.63;1.74)	0.854	1.07	(0.80;1.42)	0.645
Age									
Per one year	0.97	(0.95;0.98)	** < 0.001**	0.98	(0.97;1.00)	0.105	1.00	(0.98;1.01)	0.347
Educational level^a^									
Lower	Reference			Reference			Reference		
Higher	1.14	(0.80;1.61)	0.469	1.00	(0.60;1.67)	0.994	1.37	(1.03;1.82)	**0.033**
Chemotherapy^b^									
No	Reference			Reference			Reference		
Yes	0.48	(0.34;0.68)	** < 0.001**	1.34	(0.82;2.19)	0.248	0.82	(0.62;1.09)	0.175
Fatigue severity^c^									
No fatigue	Reference			Reference			Reference		
Mild fatigue	2.05	(1.17;3.58)	**0.012**	2.40	(0.80;7.16)	0.117	2.01	(1.34;3.01)	** < 0.001**
Moderate fatigue	3.75	(2.10;6.67)	** < 0.001**	6.40	(2.19;18.70)	** <0 .001**	4.13	(2.67;6.39)	** < 0.001**
Severe fatigue	5.22	(2.38;11.47)	** < 0.001**	7.09	(2.01;25.04)	**0.002**	2.62	(1.33;5.18)	**0.006**
Depression^d^									
No depression	Reference			Reference			Reference		
Minor depression	2.73	(1.52;4.90)	** < 0.001**	1.34	(0.58;3.10)	0.498	1.54	(0.88;2.70)	0.132
Major depression	2.50	(1.46;4.29)	** < 0.001**	2.30	(1.18;4.47)	**0.015**	1.64	(0.98;2.74)	0.061
SSUK-8 positive support									
Per step	0.95	(0.91;1.00)	0.067	0.97	(0.91;1.05)	0.476	0.99	(0.95;1.03)	0.546

	Lack of time			Physicians consider fatigue not important			Belief that there are no treatments for fatigue		
	(*N* = 980)			(*N* = 952)			(*N* = 944)		
	OR	CI	*p*	OR	CI	*p*	OR	CI	*p*
Sex									
Male	Reference			Reference			Reference		
Female	1.51	(1.12;2.05)	**0.008**	1.18	(0.86;1.63)	0.307	1.38	(0.97;1.95)	0.072
Age									
Per one year	1.01	(0.99;1.02)	0.333	1.01	(0.99;1.02)	0.324	1.01	(1.00;1.03)	0.109
Educational level^a^									
Lower	Reference			Reference			Reference		
Higher	1.14	(0.84;1.54)	0.410	0.92	(0.66;1.28)	0.614	1.03	(0.72;1.46)	0.881
Chemotherapy^b^									
No	Reference			Reference			Reference		
Yes	0.75	(0.56;1.02)	0.065	0.57	(0.41;0.80)	** < 0.001**	1.11	(0.79;1.57)	0.551
Fatigue severity^c^									
No fatigue	Reference			Reference			Reference		
Mild fatigue	2.94	(1.82;4.76)	** < 0.001**	1.90	(1.16;3.11)	**0.010**	2.66	(1.45;4.88)	**0.002**
Moderate fatigue	5.16	(3.10;8.57)	** < 0.001**	4.13	(2.48;6.89)	** < 0.001**	5.77	(3.11;10.70)	** < 0.001**
Severe fatigue	5.64	(2.73;11.67)	** < 0.001**	4.09	(1.95;8.58)	** < 0.001**	6.81	(2.99;15.52)	** < 0.001**
Depression^d^									
No depression	Reference			Reference			Reference		
Minor depression	1.98	(1.13;3.48)	**0.017**	2.34	(1.32;4.16)	**0.004**	1.24	(0.67;2.30)	0.486
Major depression	2.03	(1.20;3.43)	**0.009**	1.90	(1.11;3.25)	**0.019**	2.68	(1.56;4.60)	** < 0.001**
SSUK-8 positive support									
Per step	0.95	(0.91;0.99)	**0.021**	0.93	(0.89;0.98)	**0.003**	0.95	(0.90;1.00)	**0.032**

	Not knowing who to turn to for fatigue			Physicians’ lack of knowledge of fatigue			Perceiving ≥ 1 communication barrier^e^		
	(*N* = 963)			(*N* = 910)			(*N* = 986)		
	OR	CI	*p*	OR	CI	*p*	OR	CI	*p*
Sex									
Male	Reference			Reference			Reference		
Female	1.32	(0.98;1.77)	0.072	1.07	(0.76;1.50)	0.697	1.12	(0.84;1.50)	0.439
Age									
Per one year	1.01	(1.00;1.02)	0.078	1.01	(1.00;1.03)	0.073	0.99	(0.98;1.00)	0.084
Educational level^a^									
Lower	Reference			Reference			Reference		
Higher	0.73	(0.54;0.99)	**0.040**	0.98	(0.70;1.39)	0.923	1.34	(1.00;1.80)	**0.049**
Chemotherapy^b^									
No	Reference			Reference			Reference		
Yes	0.79	(0.59;1.07)	0.132	0.83	(0.59;1.16)	0.275	0.83	(0.62;1.11)	0.196
Fatigue severity^c^									
No fatigue	Reference			Reference			Reference		
Mild fatigue	2.06	(1.36;3.14)	** < 0.001**	2.14	(1.26;3.64)	**0.005**	2.21	(1.57;3.11)	** < 0.001**
Moderate fatigue	3.93	(2.50;6.16)	** < 0.001**	4.65	(2.68;8.07)	** < 0.001**	4.68	(3.09;7.11)	** < 0.001**
Severe fatigue	4.40	(2.11;9.15)	** < 0.001**	3.20	(1.42;7.23)	**0.005**	5.96	(2.47;14.39)	** < 0.001**
Depression^d^									
No depression	Reference			Reference			Reference		
Minor depression	2.44	(1.36;4.37)	**0.003**	1.34	(0.72;2.51)	0.358	1.79	(0.88;3.62)	0.106
Major depression	4.87	(2.65;8.98)	** < 0.001**	3.99	(2.23;7.14)	** < 0.001**	3.54	(1.50;8.37)	**0.004**
SSUK-8 positive support									
Per step	0.95	(0.91;0.99)	**0.022**	0.94	(0.90;0.99)	**0.021**	0.97	(0.92;1.02)	0.182

Bold values indicate statistical significance at the *p* < 0.05 level

For each barrier, all factors were included simultaneously in the models

^a^Lower: no degree or (lower-) secondary education degree; *Higher:* diploma qualifying for university or university degree

^b^*No:* Not having received or receiving chemotherapy or chemotherapy planned*;* Yes*:* Chemotherapy completed or currently receiving chemotherapy

^c^Referring to the Brief Fatigue Inventory (BFI) score

^d^Referring to the Patient Health Questionnaire, depression module (PHQ-9) score

^e^Based on dichotomized barrier variable

### Perceived quality of the patient–physician communication about CRF

Results concerning the patients’ perspective on the quality of patient-physician communication with regard to CRF are presented in Table 5. Of the participants who reported to be affected by CRF 27% (strongly) agreed that their exhaustion was not taken seriously by their physicians. Furthermore, 45% (strongly) disagreed with the statement ‘*My physicians are appreciative and understanding of my questions about fatigue*’. More than half of the participants who judged the respective statement (strongly) disagreed that their physicians took time to address questions about CRF. Furthermore, 28% of the respondents felt that their physicians did not feel responsible for CRF. About 40% (strongly) agreed that their physicians could give competent advice on CRF.

**Table 5:** Patients’ perceived quality of the communication with physicians about cancer-related fatigue^a^

Item	Strongly disagree		Disagree		Agree		Strongly agree	
	*n*	%	*n*	%	*n*	%	*n*	%
My exhaustion is not taken seriously by my physicians. (*N* = 627)	249	39.7	212	33.8	119	19.0	47	7.5
My physicians are appreciative and understanding of my questions about exhaustion/fatigue. (*N* = 600)	126	21.0	145	24.2	249	41.5	80	13.3
My physicians take time to address my questions about exhaustion/fatigue. (*N* = 595)	142	23.9	158	26.6	227	38.2	68	11.4
My physicians do not feel responsible for fatigue. (*N* = 574)	211	36.8	200	34.8	120	20.9	43	7.5
My physicians can give me competent advice on fatigue. (*N* = 577)	143	24.8	206	35.7	185	32.1	43	7.5

^a^These statements were only rated by the participants who sometimes, often or always felt extremely exhausted since cancer diagnosis

## Discussion

This study demonstrated gaps in the patient–physician communication about CRF and offered important insights into patient-perceived communication barriers. Our results suggest that CRF is often not discussed at all between patients and physicians. Furthermore, if a conversation about CRF takes place, patients experience the quality of communication as unsatisfactory. For example, patients reported that physicians did not feel responsible for CRF, could not give competent advice or did not respond appreciatively to questions about CRF.

Patients reported that a minority of physicians addressed CRF by asking them whether they felt exhausted. Physicians seemed more likely to raise the topic with patients who had either completed or were currently undergoing chemo-, radio- or immunotherapy. Older individuals or those with depression were, on the other hand, less likely to be asked about CRF. Besides, only 13% of the participants recalled that their treating physicians conducted a CRF screening by means of a questionnaire or a rating scale, and only one quarter of the participants received education on CRF. The quality of education about CRF did not appear to be satisfactory. These findings point out that guideline recommendations including regular, structured screenings and routine education about CRF have not yet been translated into clinical practice in Germany. Previous research conducted in different countries yielded similar results (Berger and Mooney 2016; Harrington et al. 2022; Schmidt et al. 2021). For example, in a German study, 41% of the 2,508 surveyed patients were not asked about CRF by their physicians and only 13% of those experiencing severe fatigue were screened using a questionnaire. As in the present study, older age seemed to be associated with a lower likelihood of being approached by physicians with regard to exhaustion (Schmidt et al. 2021). However, it could be that some elderly individuals were actually asked about CRF by their treating physicians but did not recall correctly due to cognitive impairment. Alternatively, since elderly people often face multimorbidity differential diagnosis is challenging and physicians might tend to normalize exhaustion, thereby bearing the risk of overlooking CRF. Similarly, due to sharing symptoms, diagnosis might also be difficult in individuals with comorbid depression (Horneber et al. 2012). Given physicians’ knowledge gaps regarding CRF one might carefully assume that some physicians avoid discussing the issue (Jones et al. 2021; Pearson et al. 2015). Thus, to increase knowledge but also confidence in dealing with CRF, specific trainings for physicians and relevant HCPs might be important. Apart from that, because of structural barriers like limited temporal and staff resources, physicians perhaps prioritize issues which are directly brought up by the patients (Jones et al. 2021; Smith et al. 2019).

Overall, the here presented results indicate that in most cases physicians do not initiate communication about CRF. But, a previous study demonstrating that half of the patients with CRF did not raise the issue with their physicians, suggests that patients too might perceive barriers making it difficult or even impeding them to address CRF in medical consultations (Stone et al. 2000). Indeed, we found that almost two thirds of cancer patients perceived ≥ 1 communication barrier, with 17% perceiving even five or more. Among those feeling impeded by exactly one communication barrier, the most frequently mentioned barriers were either of structural nature, e.g., because there was no opportunity to discuss CRF or they did not know who to turn to, or patient related, e.g., the fear of being perceived as weak. A considerable number of participants also felt time constraints in medical consultations or thought that their physicians consider CRF not to be important. Additionally, a quarter of the patients believed that their physicians do not have sufficient knowledge about CRF.

In general, the finding that a large number of patients experienced communication barriers is mostly consistent with the literature (Henselmans et al. 2015; Noordman et al. 2017). Barriers to communication and participation in medical consultations are also reported by almost half of patients with other chronic conditions such as cardiovascular diseases or diabetes (Henselmans et al. 2015). As in our study, communication barriers commonly referred not only to patients’ characteristics and HCPs’ attributes, but also to external factors (van Bruinessen et al. 2013). Few older studies explicitly dealt with communication barriers in CRF management and described that the fear of being bothersome, the belief that CRF is inevitable, the experience that the physician never raised it as an issue and time pressure as relevant barriers (Passik et al. 2002; Shun et al. 2009; Stone et al. 2000). Interestingly, these barriers coincide well with those we found in our study. It can be concluded that, despite advances in CRF research that have led to the development of specific guidelines and raised awareness of the issue, clinical practice does not seem to have improved as much as one would have expected over the past 20 years. There still appears to be an unfavorable interplay between structural, patient- and HCP-related barriers often resulting in the topic of CRF not being addressed.

In this study, we further tried to identify factors linked to the perception of communication barriers regarding CRF. We found that education has an impact on the perception of communication barriers. While participants with higher education were more likely to perceive the barrier ‘*no opportunity to discuss CRF’*, they were less likely to feel impeded by ‘*not knowing who to turn to for CRF*’. A previous, though not CRF-specific, study showed that individuals with low education and low health literacy were generally more likely to report communication barriers (Henselmans et al. 2015). Therefore, it is important to inform patients, particularly those with lower education, about responsibilities in healthcare and to explicitly encourage them to raise questions and concerns. Furthermore, major depression was related to a higher probability to report communication barriers, in particular the fear of being perceived as weak, the fear of dose reduction and the belief that there are no treatments for CRF. Depression-typical characteristics such as avolition or cognitive biases, e.g., dichotomous thinking, could enhance the perception of barriers and the feeling of being hindered by them. This finding is particularly concerning since, as mentioned earlier, physicians were reluctant to address CRF in individuals with depression. Instead, HCPs should be even more proactive in raising the topic of CRF in this subgroup.

Moreover, with regard to some barriers (e.g., ‘*physicians consider CRF not important*’, ‘*not knowing who to turn to for CRF’*) social support seemed to be protective. Similarly, in a qualitative study among cancer patients support from a companion facilitated patient participation (Henselmans et al. 2012), e.g., because caring relatives might motivate patients to take an active role in consultations or even accompany them for support. Additionally, more severe CRF was related to an enhanced perception of barriers, with regard to the global score (perceiving ≥ 1 barrier) and for all barriers separately. Based on previous studies in which patients experiencing higher levels of CRF interference with daily activities were more willing to report CRF, we expected that the distress and associated need for support resulting from stronger CRF symptoms might reduce barriers to communication (Shun et al. 2009). One possible explanation for our contrasting results could be the following: Individuals with severe CRF, who also have a strong need to discuss CRF and to receive support, are more aware of existing (structural) barriers. On the other hand, the barriers for patients with no/mild CRF, who have a lower need to address the issue, are hypothetical in nature. Their relevance might therefore be underestimated by this group.

### Clinical implications

To improve patient–physician communication about CRF, several actions are required to reduce barriers at the system, patient, and physician level. According to the framework suggested by Feldman-Stewart et al. (2005), communication is determined by needs, skills, values, beliefs, and emotions on the part of both, the patient and the physician, which mutually influence each other. In the context of CRF, knowledge gaps among physicians (skills) and a tendency to underestimate the burden caused by CRF (beliefs) might contribute to physicians being reluctant to address CRF (Jones et al. 2021; Pearson et al. 2015; Williams et al. 2016). Therefore, to increase feelings of competence and self-efficacy in dealing with CRF, physicians should be provided with the relevant information and skills in CRF-specific trainings. On the patients’ side, the fear of being perceived as weak or of being bothersome (emotions) and the belief that there are no treatments for CRF seem to be possible reasons for not bringing up CRF (Passik et al. 2002; Stone et al. 2000). Patients might not even be aware that their exhaustion is a common condition for which there is a medical term: Fatigue. Thus, measures to raise patients’ awareness of CRF and to reduce communication barriers are manifold. First, following guideline recommendations, HCPs should initiate the dialog about CRF by conducting a CRF screening, by introducing the term fatigue and by providing information about CRF (Fabi et al. 2020; National Comprehensive Cancer Network 2023). Second, a reflective communication style, i.e., taking time for explanations, using understandable everyday language instead of medical jargon, asking if everything has been understood, etc., will further make it easier for patients to process information during the medical appointment (van Bruinessen et al. 2013). Moreover, physicians should support patients to be active in consultations by encouraging them to self-monitor CRF symptoms using fatigue diaries and to prepare question prompt sheets (Henselmans et al. 2013; Milzer et al. 2022) To further promote the shift toward self-management and patient empowerment, it is also important to structure medical consultations in a way that makes patients feel comfortable, encouraged and empowered to assume an active, participatory role. For example, previous studies have described that the longer the patient-physician relationship and the more familiar they are with each other, the more willing patients are to raise own concerns (Henselmans et al. 2012). Therefore, if possible, a certain consistency should be ensured within the treatment team.

### Strengths and limitations

A strength of the present study is the large, diverse sample including numerous different cancer types. Additionally, a clearly defined phase in the cancer continuum, namely the acute phase approximately six months after diagnosis, was considered, minimizing the risk of a recall bias. Regardless, some limitations have to be noted. When looking at the recruitment flow one might notice that the participation rate was relatively low. However, from telephone conversations with patients who were invited to participate or with their relatives, we know that a considerable number did not meet the inclusion criteria (e.g., sufficient German language skills). Since patients with language barriers were excluded from study participation it can be assumed that the actual communication problem is even more pronounced in clinical practice. Furthermore, participation in the survey required effort and time which increases the likelihood that primarily motivated and interested patients were included. Thus, a selection bias cannot be excluded. In addition, only patients living in the German federal state of Baden-Wuerttemberg were included in the study, which may limit the generalizability of the findings. Moreover, our data do not allow us to draw clear conclusions about how many patients experienced communication barriers, but still addressed CRF. Additionally, we were unable to verify the validity of the self-reported data by, e.g., linking patients’ reports to those of their physicians using a matched-pairs approach. Therefore, it could be possible that more physicians raised the issue after all, but that patients did not recall it correctly. Finally, the logistic regression analyses have to be considered explorative and future studies investigating determinants of communication barriers should also take other factors such as patient activation, health literacy, and cognitive abilities into account.

## Conclusion

This study identified gaps in the patient–physician communication about CRF. Due to structural, patient-, and HCP-related barriers, CRF is often not discussed at all between patients and physicians, and when it is, the quality of communication is insufficient. To improve patient–physician communication about CRF, structural barriers need to be reduced. Furthermore, physicians should routinely address CRF and learn, e.g., in specific communication trainings, how to adopt a communication style that encourages patients to also seek a dialog about CRF and express their questions. These measures could empower patients to take an active role in CRF management.

## Data Availability

Data can be made available to scientific cooperation partners upon reasonable request.

## References

[CR1] Andrykowski MA, Schmidt JE, Salsman JM, Beacham AO, Jacobsen PB (2005) Use of a case definition approach to identify cancer-related fatigue in women undergoing adjuvant therapy for breast cancer. J Clin Oncol 27:6613–6622. 10.1200/jco.2005.07.024 10.1200/JCO.2005.07.024 PMC2562283 16170168

[CR2] Arndt V, Stegmaier C, Ziegler H, Brenner H (2006) A population-based study of the impact of specific symptoms on quality of life in women with breast cancer 1 year after diagnosis. Cancer 10:2496–2503. 10.1002/cncr.22274 10.1002/cncr.22274 17048250

[CR3] Arroll B, Goodyear-Smith F, Crengle S, Gunn J, Kerse N, Fishman T, Falloon K, Hatcher S (2010) Validation of PHQ-2 and PHQ-9 to screen for major depression in the primary care population. Ann Fam Med 4:348–353. 10.1370/afm.1139 10.1370/afm.1139 PMC2906530 20644190

[CR4] Behringer K, Goergen H, Müller H, Thielen I, Brillant C, Kreissl S, Halbsguth TV, Meissner J, Greil R, Moosmann P, Shonukan O, Rueffer JU, Flechtner HH, Fuchs M, Diehl V, Engert A, Borchmann P (2016) Cancer-related fatigue in patients with and survivors of hodgkin lymphoma: the impact on treatment outcome and social reintegration. J Clin Oncol 36:4329–4337. 10.1200/jco.2016.67.7450 10.1200/JCO.2016.67.7450 27998235

[CR5] Berger AM, Mooney K (2016) Dissemination and implementation of guidelines for cancer-related fatigue. J Natl Compr Canc Ne 11:1336–1338. 10.6004/jnccn.2016.0144 10.6004/jnccn.2016.0144 27799505

[CR6] Berger AM, Mitchell SA, Jacobsen PB, Pirl WF (2015) Screening, evaluation, and management of cancer-related fatigue: Ready for implementation to practice? CA: Cancer J Clin 3:190–211. 10.3322/caac.21268 10.3322/caac.21268 25760293

[CR7] Bower JE (2019) The role of neuro-immune interactions in cancer-related fatigue: biobehavioral risk factors and mechanisms. Cancer 3:353–364. 10.1002/cncr.31790 10.1002/cncr.31790 PMC6502236 30602059

[CR8] Campbell R, Bultijnck R, Ingham G, Sundaram CS, Wiley JF, Yee J, Dhillon HM, Shaw J (2022) A review of the content and psychometric properties of cancer-related fatigue (CRF) measures used to assess fatigue in intervention studies. Support Care Cancer 11:8871–8883. 10.1007/s00520-022-07305-x 10.1007/s00520-022-07305-x PMC9633540 36001179

[CR9] Fabi A, Bhargava R, Fatigoni S, Guglielmo M, Horneber M, Roila F, Weis J, Jordan K, Ripamonti CI, Guidelines Committee ESMO (2020) Cancer-related fatigue: esmo clinical practice guidelines for diagnosis and treatment. Ann Oncol 6:713–723. 10.1016/j.annonc.2020.02.016 10.1016/j.annonc.2020.02.016 32173483

[CR10] Feldman-Stewart D, Brundage MD, Tishelman C (2005) A conceptual framework for patient-professional communication: an application to the cancer context. Psycho-Oncol 10:801–809. 10.1002/pon.950. (**Discussion 810-801**) 10.1002/pon.950 16200514

[CR11] Harrington SE, Fisher MI, Lee JQ, Cohn J, Malone D (2022) Knowledge regarding cancer-related fatigue: a survey of physical therapists and individuals diagnosed with cancer. Physiother Theor Pr. 10.1080/09593985.2022.2056554 10.1080/09593985.2022.2056554 35353642

[CR12] Henselmans I, Jacobs M, van Berge Henegouwen MI, de Haes HC, Sprangers MA, Smets EM (2012) Postoperative information needs and communication barriers of esophageal cancer patients. Patient Educ Couns 1:138–146. 10.1016/j.pec.2011.12.004 10.1016/j.pec.2011.12.004 22244819

[CR13] Henselmans I, de Haes HC, Smets EM (2013) Enhancing patient participation in oncology consultations: a best evidence synthesis of patient-targeted interventions. Psycho-Oncol 5:961–977. 10.1002/pon.3099 10.1002/pon.3099 22585543

[CR14] Henselmans I, Heijmans M, Rademakers J, van Dulmen S (2015) Participation of chronic patients in medical consultations: patients’ perceived efficacy, barriers and interest in support. Health Expect 6:2375–2388. 10.1111/hex.12206 10.1111/hex.12206 PMC5810699 24813384

[CR15] Horneber M, Fischer I, Dimeo F, Rüffer JU, Weis J (2012) Cancer-related fatigue: epidemiology, pathogenesis, diagnosis, and treatment. Dtsch Arztebl Int 9:161 10.3238/arztebl.2012.0161 PMC3314239 22461866

[CR16] Howell D, Powis M, Kirkby R, Amernic H, Moody L, Bryant-Lukosius D, O’Brien MA, Rask S, Krzyzanowska M (2022) Improving the quality of self-management support in ambulatory cancer care: a mixed-method study of organisational and clinician readiness, barriers and enablers for tailoring of implementation strategies to multisites. BMJ Qual Saf 1:12–22. 10.1136/bmjqs-2020-012051 10.1136/bmjqs-2020-012051 33727415

[CR17] Jones G, Gollish M, Trudel G, Rutkowski N, Brunet J, Lebel S (2021) A perfect storm and patient-provider breakdown in communication: two mechanisms underlying practice gaps in cancer-related fatigue guidelines implementation. Support Care Cancer 4:1873–1881. 10.1007/s00520-020-05676-7 10.1007/s00520-020-05676-7 32793998

[CR18] Jung JY, Lee JM, Kim MS, Shim YM, Zo JI, Yun YH (2018) Comparison of fatigue, depression, and anxiety as factors affecting posttreatment health-related quality of life in lung cancer survivors. Psycho-Oncol 2:465–470. 10.1002/pon.4513 10.1002/pon.4513 28755492

[CR19] Kroenke K, Spitzer RL, Williams JB (2001) The PHQ-9: validity of a brief depression severity measure. J Gen Intern Med 9:606–613. 10.1046/j.1525-1497.2001.016009606.x 10.1046/j.1525-1497.2001.016009606.x PMC1495268 11556941

[CR20] Kuhnt S, Friedrich M, Schulte T, Cella D, Hinz A (2019) Screening properties of the diagnostic criteria for cancer-related fatigue. Oncol Res Treat 9:440–447. 10.1159/000501128 10.1159/000501128 31336371

[CR21] Löwe B, Kroenke K, Herzog W, Gräfe K (2004) Measuring depression outcome with a brief self-report instrument: sensitivity to change of the Patient Health Questionnaire (PHQ-9). J Affect Disord 1:61–66. 10.1016/s0165-0327(03)00198-8 10.1016/S0165-0327(03)00198-8 15183601

[CR22] Mendoza TR, Wang XS, Cleeland CS, Morrissey M, Johnson BA, Wendt JK, Huber SL (1999) The rapid assessment of fatigue severity in cancer patients: use of the brief fatigue inventory. Cancer 5:1186–1196. 10.1002/(sici)1097-0142(19990301)85:5%3c1186::aid-cncr24%3e3.0.co;2-n 10.1002/(sici)1097-0142(19990301)85:5<1186::aid-cncr24>3.0.co;2-n 10091805

[CR23] Milzer M, Steindorf K, Reinke P, Schmidt ME (2022) The cancer patients’ perspective on feasibility of using a fatigue diary and the benefits on self-management: results from a longitudinal study. Support Care Cancer 12:10213–10221. 10.1007/s00520-022-07397-5 10.1007/s00520-022-07397-5 PMC9559147 36227505

[CR24] Mustian KM, Alfano CM, Heckler C, Kleckner AS, Kleckner IR, Leach CR, Mohr D, Palesh OG, Peppone LJ, Piper BF, Scarpato J, Smith T, Sprod LK, Miller SM (2017) Comparison of pharmaceutical, psychological, and exercise treatments for cancer-related fatigue: a meta-analysis. JAMA Oncol 7:961–968. 10.1001/jamaoncol.2016.6914 10.1001/jamaoncol.2016.6914 PMC5557289 28253393

[CR25] National Comprehensive Cancer Network (2023) NCCN Clinical Practice Guidelines in Oncology: Cancer-related fatigue (Version 2.2023). https://www.nccn.org/login?ReturnURL=https://www.nccn.org/professionals/physician_gls/pdf/fatigue.pdf. Accessed June 13th, 2023

[CR26] Noordman J, Driesenaar JA, Henselmans I, Verboom J, Heijmans M, van Dulmen S (2017) Patient participation during oncological encounters: barriers and need for supportive interventions experienced by elderly cancer patients. Patient Educ Couns 12:2262–2268. 10.1016/j.pec.2017.06.009 10.1016/j.pec.2017.06.009 28619272

[CR27] Passik SD, Kirsh KL, Donaghy K, Holtsclaw E, Theobald D, Cella D, Breitbart W (2002) Patient-related barriers to fatigue communication: initial validation of the fatigue management barriers questionnaire. J Pain Symptom Manag 5:481–493. 10.1016/s0885-3924(02)00518-3 10.1016/s0885-3924(02)00518-3 12547048

[CR28] Pearson EJ, Morris ME, McKinstry CE (2015) Cancer-related fatigue: a survey of health practitioner knowledge and practice. Support Care Cancer 12:3521–3529. 10.1007/s00520-015-2723-8 10.1007/s00520-015-2723-8 25847296

[CR29] Revenson TA, Schiaffino KM, Majerovitz SD, Gibofsky A (1991) Social support as a double-edged sword: the relation of positive and problematic support to depression among rheumatoid arthritis patients. Soc Sci Med 7:807–813. 10.1016/0277-9536(91)90385-p 10.1016/0277-9536(91)90385-p 1948172

[CR30] Roila F, Fumi G, Ruggeri B, Antonuzzo A, Ripamonti C, Fatigoni S, Cavanna L, Gori S, Fabi A, Marzano N, Graiff C, De Sanctis V, Mirabile A, Serpentini S, Bocci C, Pino MS, Cilenti G, Verusio C, Ballatori E, NICSO (Network Italiano per le Cure di Supporto in Oncologia) (2019) Prevalence, characteristics, and treatment of fatigue in oncological cancer patients in Italy: a cross-sectional study of the Italian Network for Supportive Care in Cancer (NICSO). Support Care Cancer. 3:1041–1047. 10.1007/s00520-018-4393-9 10.1007/s00520-018-4393-9 30084104

[CR31] Schmidt ME, Scherer S, Wiskemann J, Steindorf K (2019) Return to work after breast cancer: the role of treatment-related side effects and potential impact on quality of life. Eur J Cancer Care 4:e13051. 10.1111/ecc.13051 10.1111/ecc.13051 31033073

[CR32] Schmidt ME, Hermann S, Arndt V, Steindorf K (2020) Prevalence and severity of long-term physical, emotional, and cognitive fatigue across 15 different cancer entities. Cancer Med-Us 21:8053–8061. 10.1002/cam4.3413 10.1002/cam4.3413 PMC7643651 32893501

[CR33] Schmidt ME, Bergbold S, Hermann S, Steindorf K (2021) Knowledge, perceptions, and management of cancer-related fatigue: the patients’ perspective. Support Care Cancer 4:2063–2071. 10.1007/s00520-020-05686-5 10.1007/s00520-020-05686-5 PMC7892505 32860177

[CR34] Schmidt ME, Milzer M, Weiß C, Reinke P, Grapp M, Steindorf K (2022) Cancer-related fatigue: benefits of information booklets to improve patients’ knowledge and empowerment. Support Care Cancer. 10.1007/s00520-022-06833-w 35147759 10.1007/s00520-022-06833-w PMC8853058

[CR35] Shun SC, Lai YH, Hsiao FH (2009) Patient-related barriers to fatigue communication in cancer patients receiving active treatment. Oncologist 9:936–943. 10.1634/theoncologist.2009-0048 10.1634/theoncologist.2009-0048 19738002

[CR36] Smith TG, Troeschel AN, Castro KM, Arora NK, Stein K, Lipscomb J, Brawley OW, McCabe RM, Clauser SB, Ward E (2019) Perceptions of patients with breast and colon cancer of the management of cancer-related pain, fatigue, and emotional distress in community oncology. J Clin Oncol 19:1666–1676. 10.1200/jco.18.01579 10.1200/JCO.18.01579 PMC6804889 31100037

[CR37] Stone P, Richardson A, Ream E, Smith AG, Kerr DJ, Kearney N (2000) Cancer-related fatigue: inevitable, unimportant and untreatable? Results of a multi-centre patient survey. Ann Oncol 8:971–975. 10.1023/a:1008318932641 10.1023/a:1008318932641 11038033

[CR38] Ullrich A, Mehnert A (2010) Psychometrische evaluation and validierung einer 8-Item Kurzversion der Skalen zur Sozialen Unterstützung bei Krankheit (SSUK) bei Krebspatienten. Klinische Diagnostik Und Evaluation 359:81

[CR39] van Bruinessen IR, van Weel-Baumgarten EM, Gouw H, Zijlstra JM, Albada A, van Dulmen S (2013) Barriers and facilitators to effective communication experienced by patients with malignant lymphoma at all stages after diagnosis. Psycho-Oncol 12:2807–2814. 10.1002/pon.3352 10.1002/pon.3352 23897828

[CR40] Wang XS, Zhao F, Fisch MJ, O’Mara AM, Cella D, Mendoza TR, Cleeland CS (2014) Prevalence and characteristics of moderate to severe fatigue: a multicenter study in cancer patients and survivors. Cancer 3:425–432. 10.1002/cncr.28434 10.1002/cncr.28434 PMC3949157 24436136

[CR41] Weis J, Tomaszewski KA, Hammerlid E, Ignacio Arraras J, Conroy T, Lanceley A, Schmidt H, Wirtz M, Singer S, Pinto M, Alm El-Din M, Compter I, Holzner B, Hofmeister D, Chie WC, Czeladzki M, Harle A, Jones L, Ritter S, Flechtner HH, Bottomley A, EORTC Quality of Life Group (2017) International psychometric validation of an EORTC quality of life module measuring cancer related fatigue (EORTC QLQ-FA12). JNCI J Natl Cancer I. 10.1093/jnci/djw273 10.1093/jnci/djw273 28376231

[CR42] Weis J, Wirtz MA, Tomaszewski KA, Hammerlid E, Arraras JI, Conroy T, Lanceley A, Schmidt H, Singer S, Pinto M, Alm El-Din M, Compter I, Holzner B, Hofmeister D, Chie WC, Harle A, Flechtner HH, Bottomley A (2019) Sensitivity to change of the EORTC quality of life module measuring cancer-related fatigue (EORTC QlQ-Fa12): results from the international psychometric validation. Psycho-Oncol 8:1753–1761. 10.1002/pon.5151 10.1002/pon.5151 31225669

[CR43] Williams LA, Bohac C, Hunter S, Cella D (2016) Patient and health care provider perceptions of cancer-related fatigue and pain. Support Care Cancer 10:4357–4363. 10.1007/s00520-016-3275-2 10.1007/s00520-016-3275-2 PMC4993798 27207616

